# Decreased PEDF Promotes Hepatic Fatty Acid Uptake and Lipid Droplet Formation in the Pathogenesis of NAFLD

**DOI:** 10.3390/nu12010270

**Published:** 2020-01-20

**Authors:** Kuang-Tzu Huang, Kuang-Den Chen, Li-Wen Hsu, Chao-Pin Kung, Shu-Rong Li, Chien-Chih Chen, King-Wah Chiu, Shigeru Goto, Chao-Long Chen

**Affiliations:** 1 Institute for Translational Research in Biomedicine, Kaohsiung Chang Gung Memorial Hospital, Kaohsiung 83301, Taiwan; dennis8857@gmail.com (K.-D.C.); vina920715@gmail.com (C.-P.K.); 2 Liver Transplantation Center, Department of Surgery, Kaohsiung Chang Gung Memorial Hospital, Kaohsiung 83301, Taiwan; hsuliwen1230@gmail.com (L.-W.H.); j2326311@gmail.com (S.-R.L.); clchen@cgmh.org.tw (C.-L.C.); 3 Department of Psychiatry, Kaohsiung Chang Gung Memorial Hospital, Kaohsiung 83301, Taiwan; chenfather@adm.cgmh.org.tw; 4 Division of Hepato-Gastroenterology, Department of Internal Medicine, Kaohsiung Chang Gung Memorial Hospital, Kaohsiung 83301, Taiwan; 5 Fukuoka Institute of Occupational Health, Fukuoka 815-0081, Japan; pochigoto0224@gmail.com

**Keywords:** pigment epithelium-derived factor, adipose triglyceride lipase, fatty acids, lipid droplets, CD36

## Abstract

Non-alcoholic fatty liver disease (NAFLD), the leading cause of chronic liver diseases worldwide, ranges from simple steatosis to steatohepatitis, with the risk for progressive fibrosis or even cirrhosis. While simple steatosis is a relatively benign condition, the buildup of toxic lipid metabolites can induce chronic inflammation, ultimately triggering disease progression. Pigment epithelium-derived factor (PEDF) is a secreted, multifunctional glycoprotein with lipid metabolic activities. PEDF promotes lipolysis through binding to adipose triglyceride lipase (ATGL), a key enzyme for triglyceride breakdown. In the current study, we aimed to delineate how changes in PEDF expression affect hepatic lipid accumulation. Our data revealed that hepatic PEDF was downregulated in a mouse NAFLD model. We further showed that decreased PEDF levels in hepatocytes in vitro resulted in elevated fatty acid uptake and lipid droplet formation, with concomitant upregulation of fatty acid transport proteins CD36 and fatty acid binding protein 1 (FABP1). RNA sequencing analysis of PEDF knocked down hepatocytes revealed an alteration in gene expression profile toward lipid accumulation. Additionally, decreased PEDF promotes mobilization of fatty acids, an observation distinct from blocking ATGL activity. Taken together, our data suggest that hepatic PEDF downregulation causes molecular changes that favor triglyceride accumulation, which may further lead to NAFLD progression.

## 1. Introduction

Non-alcoholic fatty liver disease (NAFLD) is highly prevalent in developed countries, affecting nearly one-third of the general population and is commonly accompanied by clinical features of metabolic syndrome including dyslipidemia, obesity, and type 2 diabetes [1,2]. Alarmingly, the incidence of NAFLD is continuously increasing among adults and especially in children because of the ongoing obesity epidemic [3]. NAFLD is characterized by the accumulation of lipids in the liver and covers a wide spectrum of liver disorders, ranging from benign simple steatosis to steatohepatitis (NASH) and advanced fibrosis and cirrhosis [4]. Although the mechanism of NAFLD progression is not completely elucidated, the current understating is that it may be an accumulative outcome of hepatic injury caused by sustained uptake and deposition of neutral lipids (mostly in forms of triglycerides). The failure of hepatocytes to metabolize excess free fatty acids and other intermediate metabolites is also a contributing factor. This increases the risk for lipotoxic tissue damage, inflammation, and distortion of liver vasculatures [5,6].

Pigment epithelium-derived factor (PEDF), a 50 kDa secreted glycoprotein, was first purified from the conditioned media of human retinal pigment epithelial cells as a neurotrophic factor [7]. Since then, PEDF has been detected in various tissues, and its diverse biological activities have been characterized. The crucial role of PEDF in lipid metabolism was first established in hepatocyte cell lines where adipose triglyceride lipase (ATGL), a major triglyceride hydrolase, prevents intracellular triglyceride accumulation [8]. ATGL specifically hydrolyzes the first ester linkage of triglycerides and is the rate-limiting enzyme in stepwise lipolysis, releasing free fatty acids from the adipose and other lipid storage tissues [9]. Clinical evidence suggests a positive correlation between circulating PEDF levels and metabolic risk factors in the obese population and patients with type 2 diabetes [10,11]. PEDF has also been shown in mouse and cellular models to direct adipocyte lipolysis and contribute to insulin resistance [12,13]. Despite its pathogenic potential, PEDF seems to play a protective role in the liver. PEDF reduces high fat diet induced obesity and NAFLD progression through its antioxidant and anti-inflammatory capacity in several animal studies [14,15,16]. However, how PEDF regulates hepatic lipid accumulation remains largely unknown.

In the current study, we found decreased expression of hepatic PEDF in a diet-induced NAFLD mouse model. Our in vitro experiments further showed that the decrease in PEDF expression resulted in an elevation in lipid accumulation, fatty acid uptake, and mobilization, with a concomitant increase in fatty acid transport proteins CD36 and fatty acid binding protein 1 (FABP1). RNA sequencing analysis of hepatic PEDF knockdown revealed alterations in gene expression toward triglyceride synthesis and lipid droplet formation. 

## 2. Materials and Methods

### 2.1. Animal Experiments

All animal experimental procedures were approved by the animal ethics committee of the Kaohsiung Chang Gung Memorial Hospital (Kaohsiung, Taiwan) (approval number: 2017121823). Eight-week-old male C57BL/6 mice (weighed approximately 20 g) were purchased from BioLASCO (BioLASCO Taiwan Co., Ltd., Taipei, Taiwan), housed in a climate-controlled room on a 12 h light/dark cycle, and allowed to acclimatize for one week ad libitum on a standard diet. These mice were then switched to a choline-deficient l-amino acid-defined (CDAA) diet (Dyets Inc.; Bethlehem, PA, USA) for 8 or 16 weeks to produce liver steatosis (*n* = 10 for each group). Standard diet was used for the same period in the control groups. The body weight was measured weekly. The animals were fasted for 6 h before sacrifice under anesthesia. Both the liver and epididymal fat deposits were dissected, weighed, and rapidly snap frozen in liquid nitrogen or fixed in 4% paraformaldehyde (PFA) for further analysis. 

### 2.2. Histological Analysis

PFA-fixed, paraffin-embedded sections (5 μm thick) were stained with hematoxylin and eosin (H&E) according to standard protocols. Tissue samples were subjected to immunohistochemical staining for PEDF and CD36. Antigen retrieval was performed by microwaving the slides in 10 mM sodium citrate buffer (pH 6.0) for 20 min, followed by incubation in 0.3% hydrogen peroxide to block endogenous peroxidase activity. The slides were then incubated overnight at 4 °C in humidified chambers with primary rabbit anti-PEDF or CD36 (Santa Cruz Biotechnology; Santa Cruz, CA, USA). Antigen–antibody complexes were detected by the avidin–biotin–peroxidase method. The slides were developed using diaminobenzidine (DAB) as a chromogenic substrate (DAKO; Carpinteria, CA, USA) and counterstained with hematoxylin.

### 2.3. Cell Culture and Treatments

The human hepatocellular carcinoma cell line Hep3B was derived from American Tissue Culture Collection (Manassas, VA, USA) and cultured in minimum essential medium (MEM) supplemented with 10% fetal bovine serum (FBS), in the presence of appropriate antibiotics and antimycotics (Biological Industries; Kibbutz Beit-Haemek, Israel). The cultures were maintained at a 37 °C, 5% CO_2_, humidified atmosphere. Atglistatin, GW6471, and GW9662 (Sigma-Aldrich; St. Louis, MO, USA) were dissolved in DMSO and added to the culture at concentrations indicated. The cells treated with the same volume of DMSO (no more than 0.1%) served as controls. Transfection with small interfering RNA (siRNA) was performed by incubating the cells with the siRNA mixture using GenMute siRNA Transfection Reagent (SignaGen Laboratories; Rockville, MD, USA) (final concentrations: 20 nM) according to manufacturer’s instructions for 6 h before switching to fresh culture media. 

### 2.4. Fatty Acid Preparation

Stock solutions (20 mM) of palmitic acid (PA) and oleic acid (OA) were complexed to fatty-acid-free bovine serum albumin (BSA) before adding to the cell culture medium. Briefly, sodium palmitate and oleate (Sigma-Aldrich; St. Louis, MO, USA) were each dissolved in 0.1 M NaOH at 65–70 °C and then mixed with 3.3 mM fatty-acid-free BSA. The mixture was then incubated at 37 °C for 1 h for conjugation. Control BSA solutions were prepared by mixing NaOH and BSA at the same concentrations, followed by incubation at 37 °C in parallel. 

### 2.5. Quantitative RT-PCR

Total RNA was isolated from cells and tissue homogenates using the RNeasy Mini Kit (Qiagen; Valencia, CA, USA) according to the manufacturer’s instructions. First strand complementary DNA (cDNA) was synthesized with 1 μg total RNA using High-Capacity Reverse Transcriptase (Applied Biosystems; Grand Island, NY, USA). Quantitative RT-PCR was performed as previously described [17]. The sequence of the primers used in this study are listed in Table S1.

### 2.6. Immunoblotting

Immunoblotting was performed as previously described [17]. Primary antibodies used in this study include: CD36 (SMφ), ATGL (F-7) (Santa Cruz Biotechnology; Santa Cruz, CA, USA), and PEDF (MAB1059) (Sigma-Aldrich; St. Louis, MO, USA). Secondary antibody was peroxidase-conjugated goat anti-mouse or rabbit IgG (1:10000, Jackson ImmunoResearch; West Grove, PA, USA). Protein bands were visualized using Immobilon Western Chemiluminescent HRP substrate (Millipore; Burlington, MA, USA) and digitalized on a G:BOX iChemi XL gel imaging system (Syngene; Cambridge, UK). Band intensity was evaluated using the ImageJ software (National Institute of Health, Bethesda, MD, USA).

### 2.7. Lipid Staining and Imaging

Lipid droplet accumulation was visualized by staining with BODIPY 493/503 (ThermoFisher Scientific; Waltham, MA, USA). The cells were first fixed in 4% paraformaldehyde for 10 min. Following appropriate washing, BOPIPY 493/503 was applied to the fixed cells (final concentration at 0.5 μg/mL) for 30 min. The cells were then counterstained with DAPI (0.5 μg/mL) to identify the nuclei and analyzed under an inverted fluorescence microscope (Olympus; Tokyo, Japan). To determine fatty acid uptake, a fluorescent palmitate analogue, BODIPY FL C16 (ThermoFisher Scientific; Waltham, MA, USA), was used. Cells were properly washed in 1% BSA and incubated in BODIPY FL C16 (final concentration at 100 nM) for 5 min before fixation. To evaluate lipid accumulation in hepatocytes, six random images at 40× magnification for each treatment condition were acquired. Lipid accumulation was presented by quantifying total fluorescence in each image divided by the cell number, both of which were quantified using the ImageJ software. 

To evaluate mobilization of fatty acids, at 24 h after siRNA transfection, cells were incubated with BODIPY 558/568 C12 (final concentration at 1 μM) (ThermoFisher Scientific; Waltham, MA, USA) for 6 h. After suitable washes, cells were then treated with OA-BSA conjugate (final concentration at 0.1 mM) for 16 h. Following fixation in 4% paraformaldehyde, lipid droplets were stained using BODIPY 493/503 (0.5 μg/mL) for 30 min. The cells were counterstained with DAPI (0.5 μg/mL) to identify the nuclei. The images were taken on the ImageXpress Micro XLS System (Molecular Devices; Sunnyvale, CA, USA) under the 10× Plan Flour objective. Nine images were captured for each experimental condition. An average of approximately 1500 cells were analyzed from each image. The intensity for the red signal of the merged images was calculated using the color threshold tool of the Image J software, followed by normalization to the cell number. 

### 2.8. RNA Sequencing Analysis

Differentially expressed genes (DEGs) were identified using RNA sequencing analysis as described previously [18]. Briefly, isolated total RNA from each sample (15 μg) was subjected to poly(A)-mRNA purification, followed by cDNA library construction using TruSeq RNA Sample Prep Kit (Illumina; San Diego, CA, USA) according to manufacturer’s instructions. Multiplexed samples were sequenced at 100 base pairs in length using the Illumina MiSeq System (Illumina, San Diego, CA, USA). For data analysis, the sequence reads were mapped to the reference genome and annotated using Strand NGS 2.1 (Strand Life Sciences; Bangalore, India). The summarized data were then assessed by statistical models (Mann–Whitney unpaired test, Benjamini–Hochberg multiple gene correction) to generate gene lists of DEGs as shown in the Supplementary Materials (Tables S2 and S3).

### 2.9. Statistical Analysis

An unpaired two-tailed Student’s t-test was used to evaluate the statistical significance between two groups. To compare between multiple measurements, statistical differences were calculated by one-way analysis of variance (ANOVA) followed by Dunn’s post hoc test using the GraphPad Prism software (GraphPad Software; San Diego, CA, USA). The results are shown as mean ± standard deviation. A value of *p* < 0.05 was considered statistically significant.

## 3. Results

### 3.1. PEDF Expression Is Decreased in a Diet-Induced NAFLD Mouse Model

**Figure 1:**
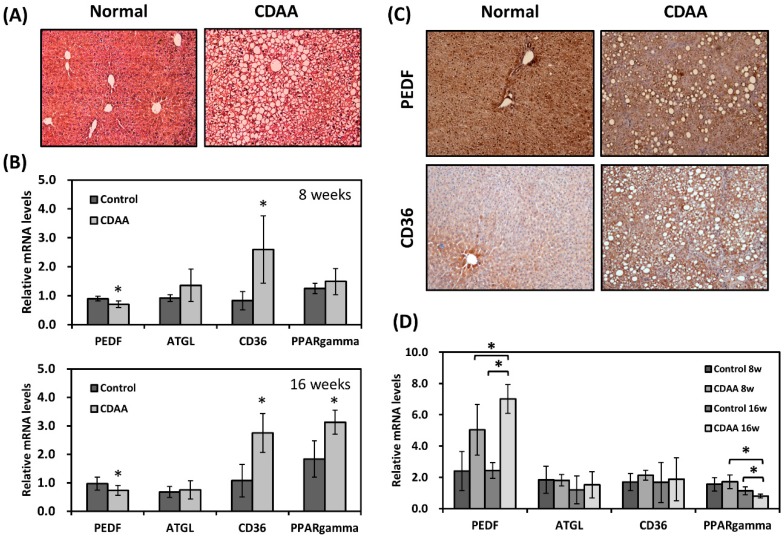
Decreased pigment epithelium-derived factor (PEDF) and increased CD36 were observed in hepatic steatosis. (**A**) Magnification: 100×. C57BL/6 mice were on a control or choline-deficient l-amino acid-defined (CDAA) diet for 8 weeks. Liver sections were stained with hematoxylin and eosin (H&E) for histological evaluation. (**B**) Hepatic gene expression was determined using quantitative RT-PCR. (**C**) Magnification: 100×. Hepatic PEDF and CD36 proteins were stained using immunohistochemical analysis. (**D**) Gene expression in the adipose tissue was determined using quantitative RT-PCR. *, Statistically significant compared with the controls at *p* < 0.05. ATGL: adipose triglyceride lipase.

PEDF deficient mice have been demonstrated to develop liver steatosis [8]. To address whether PEDF levels were aberrantly altered in liver steatosis, a CDAA diet-induced NAFLD model was employed. This model has been shown to mimic human NASH progression in both mice and rats: short-term feeding (4–8 weeks) results in isolated hepatic steatosis whereas mice on a long-term CDAA diet (16 weeks) develop dyslipidemia, steatohepatitis, and perisinusoidal/pericellular fibrosis [19,20]. In this study, the mice on CDAA diet for 8 or 16 weeks developed progressive fatty liver (Figure 1A and Figure S1A); increased α-smooth muscle actin expression and collagen deposition were also observed in the mouse liver after 16 weeks (Figure S1A,B). Quantitative RT-PCR of the liver RNA samples showed around 30% lower in PEDF expression in the CDAA liver (Figure 1B). We previously showed that CD36, a scavenger receptor that binds various ligands including long-chain fatty acids, was negatively regulated by PEDF during adipogenic differentiation [17]. Here, we also found a reciprocal correlation between PEDF and CD36 in fatty liver, as we observed higher expression (approximately threefold) in CD36 in the CDAA group compared with the controls. No significant differences were found in expression of ATGL and peroxisome proliferator-activated receptor γ (PPARγ) between the two groups at 8 weeks; however, higher levels (1.7-fold) in PPARγ was detected after 16 weeks on CDAA diet (Figure 1B). Immunohistochemical staining for PEDF showed decreased levels in the liver of the CDAA group, whereas staining for CD36 showed a moderate increase in the CDAA group, with intense staining in cells especially around areas of fat deposition (Figure 1C). PEDF expression in the adipose tissue was also examined. The CDAA diet resulted in higher expression (2.1- and 3-fold) in PEDF mRNA levels after 8 and 16 weeks, respectively (Figure 1D).

### 3.2. Decreased PEDF Is Associated with Increased Lipid Accumulation

**Figure 2:**
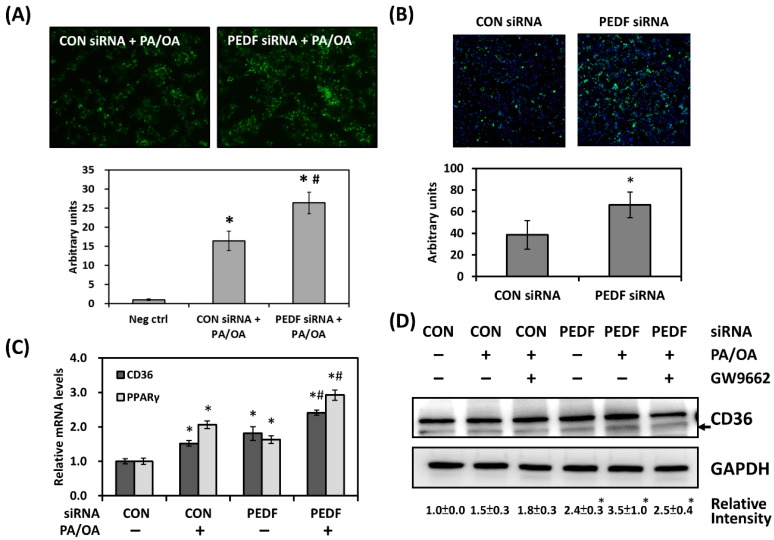
Knocking down PEDF increases lipid droplet accumulation and fatty acid uptake. (**A**) Magnification: 100×. Hep3B cells were transfected with control or PEDF siRNA, followed by incubation with vehicle or fatty acid mixture (palmitic acid (PA)/oleic acid (OA)) for 24 h. Lipid droplet accumulation was assayed by BODIPY 493/503 staining. (**B**) Magnification: 100×. Control and PEDF siRNA-transfected Hep3B cells were incubated with BODIPY FL C16 to assay fatty acid uptake. Fluorescence was then normalized to DAPI stained cell number. (**C**) Control and PEDF siRNA-transfected Hep3B cells were treated with PA/OA. Gene expression was determined using quantitative RT-PCR for CD36 and PPARγ and (**D**) immunoblotting for CD36. CD36 band intensity was quantified, normalized to that of GAPDH, and presented as mean ± standard deviation. GW9662: PPARγ inhibitor (5 μM). *, Statistically significant compared with the untreated (or control transfected, untreated) group at *p* < 0.05; #, statistically significant compared with the PEDF siRNA, untreated group at *p* < 0.05. siRNA: small interfering RNA. PPARγ: peroxisome proliferator-activated receptor γ.

To validate whether decreased PEDF expression is responsible for the increase in lipid accumulation in liver cells, we utilized an in vitro assay by incubating hepatocellular carcinoma cell line Hep3B with a mixture of free fatty acids (0.2 mM palmitate (PA) and 0.1 mM oleate (OA)). Cellular lipid droplets were fluorescently stained with BODIPY 493/503. While incubation with the PA/OA mixture resulted in lipid accumulation compared with the negative control (vehicle only, photo not shown), knockdown of PEDF in Hep3B cells further increased cellular lipid staining by about 55% (Figure 2A). A widely accepted mechanism through which PEDF deficiency leads to hepatic steatosis is decreased activity of ATGL. In addition to regulation of triglyceride hydrolysis, we suspected that PEDF may also be involved in other lipid metabolic functions such as lipid uptake and transport. By measuring the uptake of BODIPY FL C16, a fluorescent palmitate analogue, we found that Hep3B cells with PEDF knockdown exhibited higher BODIPY FL C16 fluorescence intensity when compared with control cells (Figure 2B), indicating increased fatty acid uptake. 

To determine whether the increase in fatty acid uptake was due to elevated levels of CD36 in PEDF knocked down cells, Hep3B cells were transfected with control or PEDF siRNA, followed by fatty acid (PA/OA) treatment. The fatty acid mixture moderately increased the expression of CD36 in Hep3B cells; knocking down PEDF further elevated CD36 levels (Figure 2C). This observation was corroborated by immunoblotting for CD36 protein levels (Figure 2D). Upregulation of CD36 may result from its upstream transcriptional regulator PPARγ [21], as we observed a concomitant increase of PPARγ mRNA in PEDF knocked down, fatty-acid-treated cells (Figure 2C). Treatment with an irreversible PPARγ inhibitor GW9662, however, did not significantly reverse this increase (Figure 2D).

### 3.3. Decreased PEDF Alters Cellular Fatty Acid Mobilization

**Figure 3:**
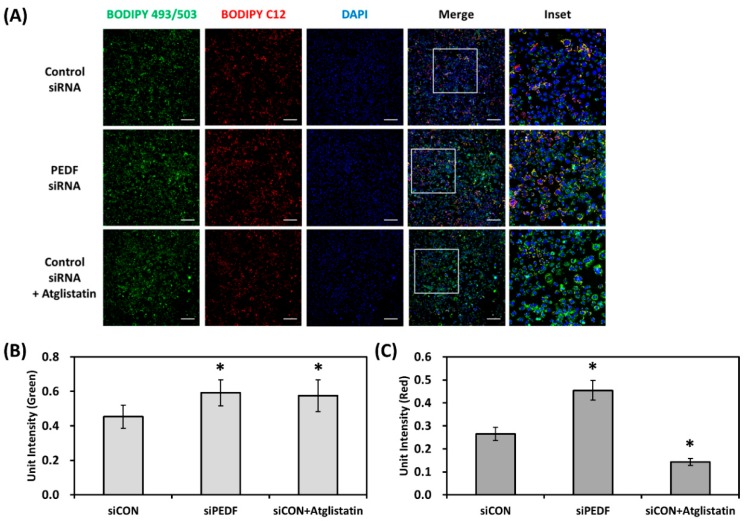
Knocking down PEDF increases hepatic fatty acid mobilization. (**A**) Magnification: 100×. Hep3B cells were transfected with control or PEDF siRNA before incubation with BODIPY 558/568 C12 (1 μM) for 6 h, followed by OA (0.1 mM) or OA + Atglistatin (25 μM) treatment for 16 h. Lipid droplets were stained with BODIPY 493/503 and nuclei were stained with DAPI. Scale bar: 200 μm. (**B**) Lipid droplet accumulation was calculated by quantification of the BODIPY 493/503 fluorescent intensity normalized to the cell number. (**C**) Mobilization of fatty acids was calculated by quantification of red signal intensity of the merged images, divided by the cell number. *, Statistically significant compared with the control transfected group at *p* < 0.05.

As we observed that PEDF knockdown increased fatty acid uptake and lipid accumulation, we next examined whether decreased PEDF expression would further affect fatty acid distribution in lipid droplets. This assay, modified from the method described by Rambold et al. and Ghosh et al. [22,23], is a pulse-chase experiment that tracks the localization of BODIPY 558/568 C12 (similar to BODIPY FL C16, with a different fluorophore and carbon chain length) in relation to lipid droplets in lipid-loaded cells. Control or PEDF siRNA transfected Hep3B cells were first labeled with a small amount of C12 (1 μM) for 6 h, followed by treatment with OA (0.1 mM) overnight. As shown in Figure 3A, the green channel area (BODIPY 493/503 panel) represents the accumulation of lipid droplets (similar to Figure 2A). We confirmed once more that knocking down PEDF increased lipid droplet accumulation (normalized to cell number) by approximately 35% (Figure 3B). The red channel intensity of the merged images, which represents the distribution of C12 fatty acids outside the lipid droplets, was also calculated and normalized to DAPI-stained cell number. Our results showed that knocking down PEDF caused a significant increase in the red channel signal by approximately 70% (Figure 3C), indicating that not only the number of lipid droplets, but also the proportion of free fatty acids not incorporated into the lipid droplets were increased in response to decreased PEDF. Interestingly, incubation with an ATGL inhibitor, Atglistatin, significantly decreased mobilization of C12 fatty acids, an observation distinct from PEDF knockdown.

### 3.4. Decreased PEDF Results in Gene Expression Changes toward Hepatic Lipid Accumulation

**Figure 4:**
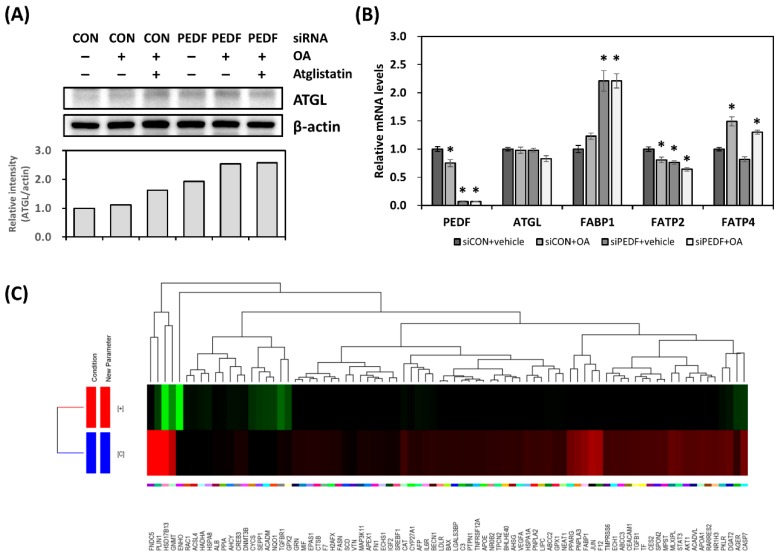
Decreased PEDF results in gene expression changes toward hepatic lipid accumulation. (**A**) Control and PEDF siRNA transfected Hep3B cells were treated with OA (0.1 mM) or OA + Atglistatin (25 μM) for 24 h. Adipose triglyceride lipase (ATGL) protein levels were determined using immunoblotting. ATGL band intensity was quantified and normalized to that of β-actin. (**B**) Gene expression was determined using quantitative RT-PCR. (**C**) Hierarchical clustering dendrogram showing non-alcoholic fatty liver disease (NAFLD)-related differentially expressed genes (DEGs) by comparing control with PEDF siRNA transfected Hep3B samples. *, Statistically significant compared with the controls at at *p* < 0.05.

With the increase in fatty acid uptake, mobilization, and triglyceride accumulation in response to PEDF downregulation, one would expect that certain gene expression changes would occur. Expression of ATGL, the PEDF binding partner, was examined. We found that the protein levels of ATGL were moderately elevated in PEDF siRNA transfected cells in the absence or presence of OA (Figure 4A). However, the mRNA levels were less affected (Figure 4B). We also measured the mRNA levels of several liver-specific fatty acid transport proteins. Fatty acid binding protein 1 (FABP1) was upregulated in PEDF siRNA transfected Hep3B cells; the expression of fatty acid transport protein 2 (FATP2) was less altered. There was no change in FATP4 expression.

To further delineate gene expression changes following PEDF knockdown, we prepared cDNA libraries from control and PEDF siRNA transfected Hep3B samples and performed mRNA sequencing. The sequencing runs generated a total of 22.67 M reads with an average length of 100 base pairs. Pre- and post-alignment quality controls were performed using the Strand NGS software (version 2.1); unaligned reads were excluded from further analysis. Differentially expressed genes (DEGs) were calculated using the DESeq method. The significance levels were corrected using Benjamini–Hochberg correction. The identified DEGs were further categorized based on the biological functions using the Gene Ontology (GO) enrichment analysis (Tables S2 and S3). We specifically searched for upregulated DEGs in response to PEDF knockdown. Among the 2258 identified DEGs, 83 were assigned in the NAFLD category. The hierarchical clustering dendrogram is shown in Figure 4C. Interestingly, several genes involved in the lipid droplet formation are listed, including perilipin 1 (*PLIN1*), diacylglycerol O-acyltransferase 2 (*DGAT2*), patatin-like phospholipase domain containing 3 (*PNPLA3*), and FABP1. Transcription factors such as sterol regulatory element binding transcription factor 1 (*SREBF1*), nuclear receptor subfamily 1 group H member 3 (*LXRα*), PPARγ, and antioxidant genes NAD(P)H quinone dehydrogenase 1 (*NQO1*), catalase (*CAT*) are also notable DEGs.

### 3.5. PEDF Is Downregulated by Fatty Acids PA and OA

**Figure 5:**
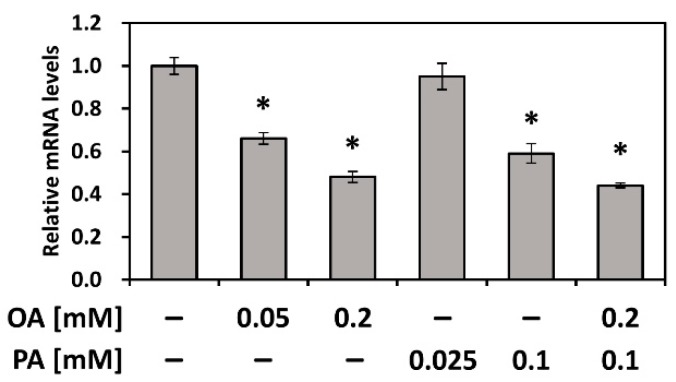
Hepatic PEDF is downregulated by fatty acids OA and PA. Hep3B cells were treated with various concentrations of fatty acids (OA, PA, or combined) for 24 h. PEDF expression was evaluated using quantitative RT-PCR. *, Statistically significant compared with the control group at *p* < 0.05.

We observed a significant decrease in PEDF expression in the NAFLD mouse model and further investigated potential mediators that negatively regulate PEDF. OA (C18:1) and PA (C16:0) are the most abundant dietary and circulatory fatty acids and are commonly used in experimental conditions [24], including our current study. Hence, we evaluated whether OA and PA affected the expression of PEDF. As shown Figure 5, we found that OA, PA, or combined treatments downregulated PEDF in Hep3B cells.

## 4. Discussion

There is a growing body of evidence that PEDF is capable of directly modulating lipid metabolism, through binding to ATGL to facilitate lipolysis. However, the function of PEDF in the liver remains largely unknown. Studies have shown that PEDF has anti-oxidative capacities and protects hepatocytes from inflammation-induced cell death during NASH progression [14,16], suggesting that PEDF is an important hepatokine beyond merely being a lipolytic agent. In this study, we demonstrate that decreased PEDF expression, observed in the mouse fatty liver, enhances fatty acid uptake and resultant lipid accumulation and mobilization in vitro, with a concomitant increase in fatty acid transport proteins CD36 and FABP1, but not FATP2 and FATP4. 

The pathogenesis of NAFLD is characterized by excessive triglyceride deposition. Notably, the majority of fatty acids to form triglyceride in the liver are derived from the adipose tissue [25]. Aberrant increase in lipolysis in the adipose tissue can cause an elevated flux of free fatty acids to the liver and increased lipid accumulation. In our study, we found that mice fed with the CDAA diet had higher expression of PEDF in the adipose tissue than the control mice, suggesting a higher lipolysis rate in the adipose tissue. This observation is in line with several high-fat-diet models [12,13], emphasizing the central role of adipose tissue lipolysis regardless of the models used. Moreover, we found that PEDF was downregulated in the CDAA mouse liver, suggesting a distinct regulatory mechanism from the adipose tissue. We further discovered that non-esterified fatty acids OA and PA, the most abundant circulatory fatty acids, are factors that can cause the downregulation, at least in vitro. Interestingly, in the mice fed with the CDAA diet, while there was 2–3-fold higher PEDF expression in the adipose tissue, only approximately 30% lower expression was observed in the liver. However, this decrease was persistent throughout the experimental period, suggesting that PEDF may exert its biological activity in the course of NAFLD progression. Indeed, PEDF has been found to be regulated by inflammasome activation in Kupffer cells and in turn to prevent hepatocyte death, an event that can lead to steatohepatitis [14]. 

In this study, we knocked down PEDF in Hep3B cells to analyze the cellular effects in response to PEDF downregulation. Our data showed that PEDF knockdown increased fatty acid uptake and triglyceride accumulation, which possibly resulted from an upregulation of fatty acid transport proteins CD36 and FABP1. CD36 is a scavenger receptor, best known for its role in the uptake of oxidized low-density lipoprotein and long-chain fatty acids [26]. Several studies have linked increased hepatic CD36 expression with elevated fatty acid uptake and lipid accumulation [27,28]. We have previously shown that decreased PEDF expression promotes differentiation of pre-adipocytes through induction of CD36 [17]. In our current work, we evaluated whether similar regulation exists in the liver. Our results showed that although this reciprocal correlation occurred both in vivo and in vitro, only a moderate increase was observed compared to the increase during adipogenic differentiation. Unlike the adipocytes, CD36 expression is low at the basal level in normal hepatocytes and hepatocellular carcinoma cell lines but can be induced with lipid-rich diets or in hepatic steatosis models. Therefore, decreased PEDF may be one of the various signals that regulate CD36 expression in vivo. Similarly, FATP2 and FATP4 are found to be upregulated in a number of high-fat-diet or genetic obesity models [29,30]. However, in our in vitro experiments, PEDF knockdown slightly decreased FATP2 levels and had no effect on FATP4 expression. FABP1, on the other hand, is an abundant protein in the liver and may play bigger roles in PEDF-mediated effects. FABP1, the liver-specific intracellular carrier of a broad range of hydrophobic ligands, is crucial in fatty acid trafficking and compartmentalization [31]. It has also been demonstrated by Wolfrum et al. and several later studies that FABP1 is responsible for the uptake of long-chain fatty acids [32]. Our observation of increased fatty acid uptake can be a combination of these expression changes. 

The upregulation of FABP1 following PEDF knockdown suggests possible alterations in fatty acid trafficking. Triglyceride synthesis, lipase activity, and lipid signaling all affect mobilization of fatty acids [33]. This is especially important as excess free fatty acids and lipid metabolites can be harmful to the cells. Our mRNA sequencing results suggest that several genes that promote lipid droplet formation are upregulated in Hep3B cells transfected with PEDF siRNA. In the in vitro study, we found that in PEDF knocked down Hep3B cells, ATGL protein levels were increased while the mRNA was little affected, suggesting that regulation of ATGL by PEDF was mainly at the protein level. This is consistent with the previous findings by us and others that during adipogenic differentiation, PEDF induces ATGL-mediated lipolysis and increases ATGL protein turnover, a possible feedback mechanism [12,17]. It has been recently identified that the E3 ubiquitin ligase for ATGL is COP1 [22], which also controls the level of fatty acid synthase and FoxO1 transcription factor, further strengthening the crucial role of PEDF–ATGL action in lipid metabolism. 

In the fatty acid mobilization assay, although the total lipid droplet accumulation was increased in PEDF knocked down, lipid-loaded cells, these cells had more diffused red fluorescence, indicating less incorporation into lipid droplets. This observation is consistent with a recent study by Niyogi et al., showing increased fatty acid mobilization in PEDF knocked down HepG2 cells [34]. However, in their study, knocking down PEDF decreased triglyceride accumulation, which is different from our results and a general understanding of PEDF being a lipolytic stimulant. Our results also showed that by blocking ATGL activity with the Atglistatin inhibitor, total lipid droplet accumulation was increased, an effect similar to PEDF knockdown. Nevertheless, the red fluorescent signal mostly retained in the lipid droplet, suggesting that PEDF may affect redistribution of fatty acids through additional mechanisms other than through ATGL. 

As mentioned above, hepatic PEDF expression can be regulated by inflammasome activation during the progression toward steatohepatitis [14]. Conversely, the authors found that monocyte-derived macrophages have an opposing effect on PEDF expression. In our study, we aimed to search for potential mediators that regulate PEDF at an earlier stage (i.e., before apparent inflammation occurs). Our data suggest that dietary fatty acids OA and PA downregulate PEDF expression. Various fatty acids are known PPAR ligands. Using the JASPAR CORE database [35], we found two putative response elements for PPARα binding in the promoter region of PEDF (at −1342 and −78, respectively). Our preliminary data showed that co-treatment with OA and an PPARα inhibitor GW6471 reversed the effect of OA (not shown), indicating a PPARα-dependent mechanism in regulating PEDF expression. Interestingly, PPARα activation also facilitates transcription of multiple genes involved in fatty acid uptake, intracellular transport, and oxidation [36,37], which adds another level of regulation. 

One limitation of our current research is that although this study provides new perspective on PEDF activity in the liver (e.g., tissue-specific regulation, involvement in fatty acid uptake, and distribution), whether these observations can be extrapolated to human is not known. For example, results from several rodent models of obesity suggest increased PEDF expression in the adipose tissue [12,38]. However, this is in contrast to the findings in a recent human study demonstrating that adipose tissue PEDF is decreased in obese to morbidly obese subjects and is not associated with the circulating levels [39]. Therefore, further studies are needed to explain these discrepancies.

## 5. Conclusions

In this study, we demonstrate a moderate but significant decrease in hepatic PEDF expression in NAFLD mice, in contrast with elevated expression in the adipose tissue, suggesting tissue-specific regulation. Our data also indicate that downregulation of hepatic PEDF in vitro results in changes in gene expression profile toward increased fatty acid uptake and triglyceride accumulation. Interestingly, an alteration in intracellular fatty acid mobilization was observed in response to decreased PEDF. This may be crucial as free fatty acids released from the lipid droplets can be toxic to the cells. Further studies will include investigation on whether downregulation of PEDF affects fatty-acid-induced lipotoxicity, endoplasmic reticulum stress, and inflammatory response. Data from the mRNA sequencing comparing control with PEDF knocked down cells also reveal a number of cellular processes of interest including lipid droplet formation, lipogenesis, and insulin signaling. We hope that these results will provide more insight for the involvement of PEDF in the pathogenesis and progression of NAFLD.

## Supplementary Materials

The following are available online at https://www.mdpi.com/2072-6643/12/1/270/s1, Figure S1: Mice on a CDAA diet develop hepatic fibrosis, Table S1: List of primers used in this study, Table S2: Subnetwork analysis of 2258 upregulated DEGs (siPEDF vs. siCON), Table S3: Subnetwork analysis of 2473 downregulated DEGs (siPEDF vs. siCON).
