# Supplementary material for: Decreased PEDF Promotes Hepatic Fatty Acid Uptake and Lipid Droplet Formation in the Pathogenesis of NAFLD
Source: Nutrients. 2020 Jan 20;12(1):270. doi: 10.3390/nu12010270 (PMC7019565; doi:10.3390/nu12010270)
Supplement: Supplementary file 1 [file nutrients-12-00270-s001.zip › Figure S1 Table S1.pdf]

## Supplementary Materials

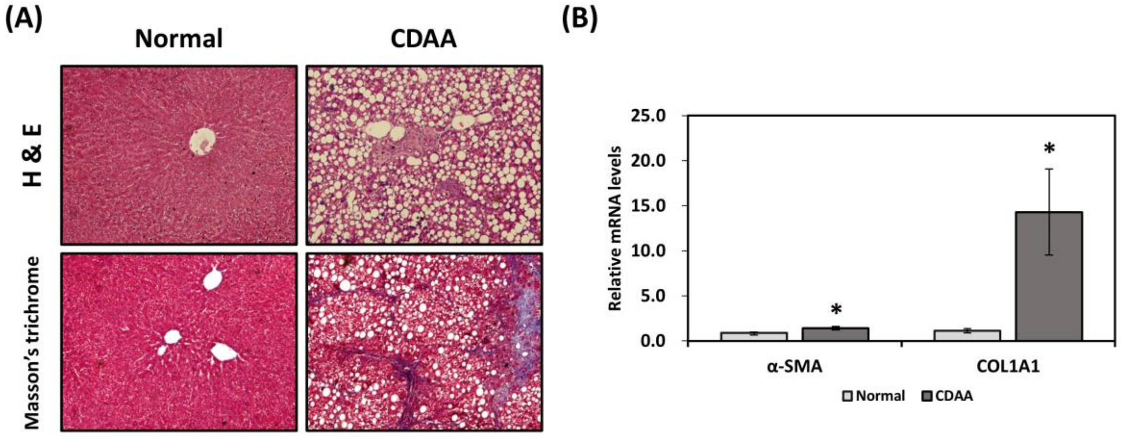


**Figure S1.** Mice on a CDAA diet develop hepatic fibrosis. (**A**) C57BL/6 mice were fed with a control or CDAA diet for 16 weeks. Liver sections were stained with H & E for histological evaluation and Masson's trichrome for collagen deposition. (**B**) Hepatic gene expression of $\alpha$-smooth muscle actin ($\alpha$-SMA) and collagen 1$\alpha$1 (COL1A1) was determined using quantitative RT-PCR. *, statistically significant compared with the normal diet group at $p<0.05$.

**Table S1.** List of primers used in this study.

| Gene name | Species | Forward primer | Reverse primer |
|---|---|---|---|
| PEDF | Human | CGACCAACGTGCTCCTGTCT | GATGTCTGGGCTGCTGATCA |
| ATGL | Human | CAACGCCACGCACATCTAC | CAATGAACTTGGCACCAGCC |
| PPARγ | Human | GATGACAGCGACTTGGCAAT | TGAGGACTCAGGGTGGTTCA |
| CD36 | Human | GTGATGATGAACAGCAGCAACA | CCTCAGCGTCCTGGGTTACA |
| FABP1 | Human | GCTGGGTCCAAAGTGATCCA | TGTCACCTTCCAACTGAACCA |
| FATP2 | Human | AGCGGATTGAAGGCAGATGA | AGTCCGCAAGGCAAGAGTAG |
| FATP4 | Human | AAGCACAGAACACCTGGACC | GTGGTGCCGGATGTGTAGAT |
| β-actin | Human | TCTGGCACCACACCTTCTACAATG | AGCACAGCCTGGATAGCAACG |
| PEDF | Mouse | ACGATACGGCTTGGACTCTG | GTCAAGTTCTGGGTCACGGT |
| ATGL | Mouse | GCCAACGCCACTCACATCTA | AATGTTGGCACCTGCTTCAC |
| PPARγ | Mouse | AGAGGGCCAAGGATTCATGACCAGG | TTCAGCTTGAGCTGCAGTTCCAGGG |
| CD36 | Mouse | TGGCCAAGCTATTGCGACAT | TTCAGATCCGAACACAGCGT |
| α-SMA | Mouse | GACAGGATGCAGAAGGAGATCAC | AGCCACCGATCCAGACAGAGT |
| COL1A1 | Mouse | CGTCTGGTTTGGAGAGAGCAT | TGGACATTAGGCGCAGGAA |
| β-actin | Mouse | TGTCCACCTTCCAGCAGATGT | AGCTCAGTAACAGTCCGCCTAGA |
